# Dietary behaviors, lifestyle habits, food insecurity risk and depressive symptoms among New York City adolescents: a cross-sectional study

**DOI:** 10.1186/s12887-026-06819-1

**Published:** 2026-04-23

**Authors:** Jaynisha Jackson, Teresa Conigliaro, Jordan Fenlon, Elizabeth Solomon, Erin A. Dowling, Nicole Dreisbach, Meghan Hamwey

**Affiliations:** 1 Bureau of Children, Youth, Families, and Developmental Disabilities, New York City Department of Health and Mental Hygiene, Long Island City, NY 11101 USA; 2 Bureau of Chronic Disease Prevention, New York City Department of Health and Mental Hygiene, Long Island City, NY 11101 USA

**Keywords:** Self-reported depressive symptoms, Dietary behaviors, Lifestyle habits, Food insecurity risk, New York City, Students

## Abstract

**Background:**

Adolescence is a developmental period marked by changes that increase susceptibility to mental health problems. Rising rates of depression among United States adolescents underscore the need to identify contributing factors. Dietary behaviors, lifestyle habits, and food insecurity have been linked to poor mental health outcomes, with structural and systemic inequities shaping access to healthy behaviors. Therefore, we examined the association between self-reported depressive symptoms with dietary behaviors, lifestyle habits, and food insecurity risk among New York City (NYC) public high school students, and variations within and across racial and ethnic groups.

**Methods:**

Data from the 2023 NYC Youth Risk Behavior Survey was utilized. Bivariate analyses assessed differences in self-reported depressive symptoms, dietary behaviors, lifestyle habits, and food insecurity risk across racial and ethnic groups and examined how these factors were associated with self-reported depressive symptoms. Logistic regression models controlling for sex, sexual identity, gender identity, age group, and borough of residence assessed these associations in the overall sample and within racial and ethnic groups.

**Results:**

Higher prevalences of not eating fruit or vegetables, sugary drink consumption, not eating breakfast, not participating in physical activity, and being at risk for food insecurity were seen among Black and Latino/a students. However, associations between self-reported depressive symptoms with dietary behaviors, lifestyle habits, and food insecurity risk varied within racial and ethnic groups. Despite Black students having the highest prevalence for food insecurity risk and sugary drink consumption, neither was associated with self-reported depressive symptoms. Food insecurity risk was associated with increased odds of self-reported depressive symptoms among White (AOR 2.42; 95% CI 1.21–4.85), Latino/a (AOR 2.09; 95% CI 1.50–2.90), Asian (AOR 1.83; 95% CI 1.04–3.23) and students of another race (AOR 3.04; 95% CI 1.11–8.34). The absence of associations among Black students warrants further research into potential underlying factors.

**Conclusion:**

Dietary behaviors, lifestyle habits, and food insecurity risk are differentially associated with self-reported depressive symptoms within racial and ethnic groups. High prevalences of insufficient sleep and food insecurity risk highlight those as key targets for intervention. Addressing these factors, alongside upstream social determinants is necessary for promoting mental health equity among NYC public high school students.

**Supplementary Information:**

The online version contains supplementary material available at 10.1186/s12887-026-06819-1.

## Background

Adolescence is a developmental period characterized by physical, psychological, and hormonal changes that shape how adolescents feel, think, and interact with those around them [1, 2]. These changes can make adolescents more susceptible to mental health problems [3, 4]. Within the United States (US) in 2023, approximately 20% of adolescents had a current, diagnosed mental or behavioral health condition, a 35% increase from 2016 [5]. Furthermore, from 2013 to 2023, the prevalence of depression among adolescents increased from 8.2% to 13.1% [6]. Such increases are alarming since mental health conditions are associated with difficulties at school, partaking in risk behaviors, and suicide [3].

Dietary behaviors have been shown to be associated with and theorized to impact adolescent mental health [7]. The unique social and behavioral changes and rapid development that accompany adolescence make it a critical time for adopting healthy behaviors that support future health [8]. However, based on What We Eat in America, National Health and Nutrition Examination Survey data, US adolescents have the lowest healthy eating index scores across all age groups [9]. Fewer than 2% of adolescents ages 14 to 18 meet the recommended intake of vegetables and only 11–23% of individuals 9 years or older meet the recommended intake of fruit [10]. Most US adolescents consume above the recommended limits of added sugars, saturated fat, and sodium, nutrients associated with health harms [9]. These eating behaviors (along with skipping breakfast and consuming caffeine) have been associated with psychological distress, unhappiness, perceived symptoms of depression and stress, loneliness, and greater severity of anxiety [11–14]. In addition, consumption of ultra-processed food is high [15] and is associated with increased risks of depression and anxiety, both directly and indirectly through its association with health conditions, such as obesity, that may also elevate these risks [16–21]. While the exact mechanisms of the associations between dietary intake and mental health outcomes are not clear, evidence suggests that diets high in refined sugar, refined carbohydrates, and low in healthy fats may contribute to inflammation, high glycemic load, and issues with the gut microbiome, health conditions that can be associated with increased risk of negative mental health outcomes [22–26].

Similarly, lifestyle habits such as physical activity and sleep can affect adolescent mental health [27]. Research shows that only 1 in 4 high school students engaged in the recommended amount of physical activity per day (at least 60 min) or obtained the recommended amount of sleep on an average school night (at least eight hours) [28]. Both insufficient sleep duration and physical inactivity have been linked to increased risk of depression and anxiety in children and adolescents [29]. For insufficient sleep, this may be due to disruptions to emotional control in the brain [30]. Meanwhile, a lack of physical activity may mean that individuals are missing out on the benefits of mood-boosting neurotransmitters, distractions from negative thoughts [31, 32] and improved sleep duration [29]. There may also be multidirectional relationships between these factors, indicating that insufficient sleep, physical inactivity and depressive symptoms may influence and/or exacerbate one another [33, 34].

Food insecurity, defined as a household-level economic and social condition of limited or uncertain access to adequate food, is associated with both physical and mental health [35, 36]. Food insecurity is associated with an increased risk of chronic conditions [37, 38] and food-insecure households often rely on low-cost, energy-dense unhealthy foods (e.g., ultra-processed foods), skip meals, or reduce portions [36], which can exacerbate chronic conditions like diabetes and hypertension [39, 40]. Research that utilized data from adolescents across multiple countries and diverse populations showed that food insecurity is associated with higher odds of poor mental health, suicidal ideation, chronic worrying, stress, depression, anxiety, and chronic loneliness among adolescents when compared to their food-secure peers [41, 42].

Diet quality, lifestyle habits, and food insecurity are impacted by social determinants of health, which inform one’s ability to access, purchase, and prepare healthy food and live in a safe physical environment. These determinants include but are not limited to income, occupation, educational attainment, and geographic location and can drive unhealthy behaviors through sometimes intangible, systemic, environmental, and social factors beyond individual choices [43, 44]. Historic and current policies, systems, and structural factors, such as systemic racism, fundamentally shape the environments and conditions that either inhibit or promote healthy behaviors.

New York City’s (NYC) data and unique landscape demonstrate the impact of these upstream drivers. In 2022, approximately 1.9 million New Yorkers were living in poverty, with 53% of adults in this population experiencing food insecurity [45]. Low food security was more common among Black (24%) and Latino (29%) NYC adults living in poverty compared to their White counterparts (13%) and high food security was less likely among those in households with children (24%) compared with those in households without (32%) [45]. Further, of NYC adults living in poverty, those who reported symptoms of depression were twice as likely, and those who reported symptoms of anxiety were nearly twice as likely, to experience very low food security compared with those without these symptoms [45]. Several NYC neighborhoods, many of which are predominately Black, Latino, and low-income, have limited access to full-service grocery stores [46, 47]. Bodegas, or corner stores, are more common than full-service grocery stores in neighborhoods with higher levels of poverty [48]. More bodegas than supermarkets may indicate a food environment with limited healthy food options, as bodegas tend to offer more ultra-processed foods compared to supermarkets. Additionally, residents in urban cities report barriers to cooking at home including time constraints and limited cooking equipment, space, preparation, and cooking skills [46]. Finally, while NYC communities with lower incomes may have access to urban green spaces, those spaces were on average smaller than spaces in other communities [49].

This unique landscape also influences mental health and reinforces inequities and disparities. In 2023, 11% of NYC adults were diagnosed with a major depressive disorder or severe depression and 8% had serious psychological distress (SPD) [50]. Black, Latino, Middle Eastern or North African, and multi-racial adults were more likely to report SPD than their White counterparts [50]. In a 2023 survey, 11% of teens reported severe depression symptoms [50]. Additionally, 18% of public high school students in NYC reported suicide ideation and 14% reported suicide attempt in 2023, with the highest prevalences seen among Black, Latino, and Another or multi-race students [51]. Neighborhood factors such as high poverty levels and witnessing or experiencing neighborhood violence are attributed to higher rates of SPD [50]. Households with lower income, experiencing food insecurity, and economic hardship have also been associated with higher rates of SPD [50, 52].

Addressing inequities in adolescent mental health and understanding the factors contributing to these outcomes is essential, as these mental health conditions can extend into adulthood, creating long-term morbidity and lowering life satisfaction [3, 53, 54]. While these relationships have been studied using national data, we sought to investigate how dietary behaviors, lifestyle habits and food insecurity risk relate to self-reported depressive symptoms among NYC public high school students. Further, we explore how these relationships vary within and across racial and ethnic groups. We hypothesized a priori that poor dietary behaviors (e.g., not consuming vegetables or fruit, sugary drink consumption, not eating breakfast, and processed meat consumption), adverse lifestyle habits (e.g., not participating in physical activity and insufficient sleep), and being at risk for food insecurity would be associated with higher odds of self-reported depressive symptoms. Finally, we hypothesized that these associations would differ within and across racial and ethnic groups.

## Methods

### Data source

This study utilized data from the 2023 NYC Youth Risk Behavior Survey (NYC YRBS), an anonymous school-based cross-sectional survey administered to NYC public high school students in grades 9–12. This 99-item survey covered various topics including mental health, dietary behaviors, physical activity, and drug and alcohol use [55]. The NYC YRBS is part of the Centers for Disease Control and Prevention’s (CDC) nationwide Youth Risk Behavior Surveillance System and is an ongoing collaboration with the NYC Health Department and NYC Public Schools (NYCPS).

The NYC YRBS is conducted biennially and employs a stratified two-stage cluster design to produce a representative sample of NYC public high school students [55]. In the first stage, schools are randomly selected with probability proportional to a school’s enrollment size [55]. In the second stage, classrooms are placed in a classroom-level sample frame then randomly selected [55]. Students in the selected classrooms are eligible to participate in the NYC YRBS but have the choice to opt out. Completed surveys are sent to the CDC and datasets are cleaned and edited for inconsistencies [56]. A weighting factor is applied to each response to adjust for nonresponse with final weights scaled to match the NYC public high school population [55]. Data presented in this article are the weighted population estimates.

In 2023, the survey was completed by 8,338 students from 77 public, charter, and vocational high schools across the city. There was a 77% school response rate, 73% student response rate, and 57% overall response rate.

### Measures

The primary mental health outcome was self-reported depressive symptoms. Self-reported depressive symptoms were measured by the question “During the past 12 months, did you ever feel so sad or hopeless almost every day for 2 weeks or more in a row that you stopped doing some usual activities?” (yes or no).

Supplemental Table 1 describes the questions and analytic coding for the exposures included in this analysis. Exposures were four self-reported dietary behaviors which included two questions to calculate vegetable and fruit consumption, two questions to calculate sugary drink consumption, and one question each to measure breakfast consumption and processed meat consumption. Processed meats and sugary drinks were included as proxies for ultra-processed foods [57]. These variables are indicators of dietary patterns rather than precise measures of dietary intake or dietary quality. Lifestyle habits were measured by one question each about daily physical activity and daily sleep, and two measures were used to determine food insecurity risk.

Single item measures were standard YRBS questions, with most of the 2023 questions demonstrating substantial reliability during a recent test-retest study [56]. However, composite variables (e.g. vegetable and fruit consumption, sugary drink consumption) were created for this analysis, and were not validated. Food insecurity risk was based on the two-item food insecurity screener Hunger Vital Signs [58]. This screener was later validated for use among youth [59].

### Demographics

Demographic variables included sex (male or female) and borough of residence (The Bronx, Brooklyn, Manhattan, Queens, and Staten Island). Age group was based on the self-reported age item with the response options of 12 years old or younger, 13 years old, 14 years old, 15 years old, 16 years old, 17 years old, and 18 years old or older. For this analysis, responses were recoded into the four age group categories: 12 years or younger to 14 years old, 15 years old, 16 years old, and 17 years old or older to create similar outcome distributions within each category and account for small sample sizes at the youngest and oldest age ranges.

Race and ethnicity were assessed using the two questions: (1) “Are you Hispanic or Latino?” (yes or no) and (2) “What is your race? (Select one or more responses)” (American Indian or Alaska Native, Asian, Black or African American, Middle Eastern or North African, Native Hawaiian or Other Pacific Islander, White, Another race not listed). Students who responded “yes” to being Hispanic or Latino were coded as Latino/a, regardless of race selection. The NYC Health Department follows CDC YRBS methodology for Hispanic/Latino classification, but in our reporting, the category is labeled Latino/a instead of Hispanic/Latino [56]. Among non-Latino/a students, race was categorized as White (including those who identified as Middle Eastern or North African), Black, Asian, and students of another race. The categorization of Middle Eastern or North African students with White is based on the Office of Management and Budget Statistical Policy Directive No. 15, the federal standard for race and ethnicity data collection used by YRBS [60]. Students of another race included those who identified as American Indian/Alaska Native, Native Hawaiian/Other Pacific Islander or Multi-racial.

For sexual identity, responses were dichotomized into heterosexual or sexual minorities. Sexual minorities included students who described themselves as gay or lesbian, bisexual, described their sexual identity some other way, or questioned their sexual identity. Similarly, gender identity was dichotomized into cisgender or gender minority. Gender minorities included students who identified as transgender girl or woman, transgender boy or man, non-binary, identified some other way, or questioned their gender identity.

### Missingness of data

Overall missingness was assessed for demographics, exposures, and the outcome. Among demographics, missingness ranged from 1% for age, 2% for sex, 3% for borough of residence, 4% for race/ethnicity, 8% for sexual identity, and 17% for gender identity. Among exposures, missingness ranged from 17% for vegetable and fruit consumption, 18% for processed meat consumption, 19% for breakfast consumption, 20% for sugary drink consumption, 20% for physical activity, 26% for daily sleep, and 29% for food insecurity risk. The outcome, self-reported depressive symptoms had 3% missingness.

Logistic regression analyses were conducted using a complete-case approach where participants with missing data on the outcome or exposures were excluded. This resulted in a final analytic sample of 4,141 participants, highlighting a substantial reduction from the overall study population. Adjusted chi-square tests comparing the included and excluded samples, considering the complex survey design, suggest that participants with missing data were more likely to be male, Latino/a or Black, reside in the Bronx, and identify as heterosexual or a gender minority. Participants with data missing on the exposures were more likely not to eat vegetables or fruit (46.3% vs. 37.0%), drink a sugary drink one or more times per day (39.2% vs. 34.4%), eat processed meat one or more times per day (43.1% vs. 36.7%), not participate in at least 60 min of physical activity (40.5% vs. 34.2%) and be at risk for food insecurity (36.6% vs. 23.2%). Missingness in the outcome did not vary between the included and excluded sample.

### Statistical analysis

All analyses were conducted using SAS-callable SUDAAN Statistical Software (version 11.0.4) and accounted for the complex survey design by incorporating sample weights and adjusting for stratification and clustering by the provided stratum and primary sampling unit variables. These survey design variables were also incorporated to account for unequal probabilities of selection [61].

Descriptive statistics were calculated to describe the distribution of student characteristics for the overall study population. A series of analyses were conducted to examine the association between self-reported depressive symptoms, dietary behaviors, lifestyle habits, and food insecurity risk. First, chi-square (*Χ*^*2*^) tests were used to assess bivariate differences in self-reported depressive symptoms, dietary behaviors, lifestyle habits, and food insecurity risk across the racial and ethnic groups. An additional chi-square test was performed within each racial and ethnic group to explore bivariate associations with self-reported depressive symptoms as an outcome with dietary behaviors, lifestyle habits, and food insecurity risk. Assumptions of chi-square, including adequate cells counts (≥ 5) and independence of observations (addressed via survey design variables) were met.

Multivariable logistic regression models were subsequently generated for the overall population and stratified by race and ethnicity to understand the association between self-reported depressive symptoms with dietary behaviors, lifestyle habits, and food insecurity risk. Assumptions for logistic regression, including independence of observations (addressed via survey design variables) and absence of multicollinearity, were met. Multicollinearity among exposures and covariates were assessed using variance inflation factors (VIFs) for stratified models. All VIFs were < 2, indicating no evidence for multicollinearity.

Analyses were stratified by race and ethnicity to assess whether associations differed across groups based on prior research documenting differences in lifestyle behaviors, food insecurity , and mental health outcomes across racial and ethnic groups [45, 51, 62–64]. To further justify stratification, models were run testing the interaction between race and ethnicity and dietary behaviors, lifestyle habits, and food insecurity risk. A significant interaction was only observed between drinking a sugary drink one or more times per day and race and ethnicity (*p* = 0.031). The final models were controlled for age group, sex, borough of residence, sexual identity, and gender identity and results from these models were reported as adjusted odds ratios (AORs). Age group and sex were included as common demographic confounders, given their established associations with mental health, dietary behaviors, lifestyle habits, and food insecurity. Sexual and gender identity were included as prior research has shown that sexual and gender minority youth differ from their peers in risk for depression, food insecurity, dietary behaviors, and lifestyle habits [65–70]. Borough of residence was included because neighborhood characteristics such as housing qualities, access to resources (e.g., grocery stores, convenience stores, parks, playgrounds), poverty level, and safety concerns have been found to be associated with depression, dietary behaviors, lifestyle habits, and food insecurity [71–75].

All statistical tests were considered statistically significant if *p* < 0.05 and all estimates were reported with corresponding 95% confidence intervals (CIs). Estimates with a relative standard error greater than 30%, the 95% confidence interval half width was greater than 10, or the sample size was too small were considered unreliable and were flagged to be interpreted with caution.

## Results

Table 1 shows the distribution for each weighted characteristic of student participants. The distribution across the four age groups was 26.0% <12 years to 14 years old, 24.7% 15 years old, 24.0% 16 years old, and 25.3% 17 years or older. Approximately half of the students were female (49.1%). The race and ethnicity distribution among students included 40.5% Latino/a, 24.9% Black, 17.3% Asian, 13.3% White, and 4.1% students of another race. Almost a third of students (32.2%) resided in Queens, 27.5% in Brooklyn, 21.4% in The Bronx, 10.5% in Manhattan, and 8.4% in Staten Island. Most students were heterosexual (75.2%) and 24.8% identified as a sexual minority. Most students (88.1%) identified as cisgender. More than a third of students (35.5%) reported depressive symptoms.

**Table 1:** Weighted characteristics of New York City (NYC) public high school students

Characteristics	Weighted *N *(rounded)	Weighted % (95% CI)
Age group		
12 years or younger to 14 years old	69000	26.0 (21.6–30.9)
15 years old	66000	24.7 (21.0-28.8)
16 years old	64000	24.0 (20.8–27.5)
17 years old or older	67000	25.3 (21.6–29.4)
Sex		
Female	129000	49.1 (45.5–52.8)
Male	133000	50.9 (47.2–54.5)
Race/Ethnicity		
White	34000	13.3 (10.0-17.5)
Black	64000	24.9 (21.0-29.1)
Latino/a	104000	40.5 (35.7–45.5)
Asian	44000	17.3 (13.2–22.3)
Another race	10000	4.1 (3.6–4.6)
Borough of residence		
The Bronx	56000	21.4 (18.4–24.8)
Brooklyn	71000	27.5 (21.2–34.9)
Manhattan	27000	10.5 (7.7–14.2)
Queens	84000	32.2 (27.7–37.0)
Staten Island	22000	8.4 (6.1–11.5)
Sexual identity		
Heterosexual (straight)	185000	75.2 (72.5–77.7)
Sexual minorities	61000	24.8 (22.3–27.5)
Gender identity		
Cisgender	195000	88.1 (83.3–91.6)
Gender minorities	26000	11.9 (8.4–16.7)
Self-reported depressive symptoms^a^		
Yes	92000	35.5 (32.5–38.6)
No	167000	64.5 (61.4–67.5)
Did not eat vegetables or fruit^b^		
Yes	21000	9.5 (7.4–12.1)
No	200000	90.5 (87.9–92.6)
Drank a sugary drink one or more times per day^c^		
Yes	65000	30.6 (27.8–33.5)
No	148000	69.4 (66.5–72.2)
Did not eat breakfast^d^		
Yes	44000	20.4 (18.4–22.6)
No	172000	79.6 (77.4–81.6)
Ate processed meat one or more times per day^e^		
Yes	27000	12.4 (11.1–13.8)
No	192000	87.6 (86.2–88.9)
Did not participate in at least 60 min of physical activity^f^		
Yes	43000	20.1 (17.0-23.7)
No	170000	79.9 (76.3–83.0)
Insufficient sleep^g^		
Yes	158000	80.2 (78.2–82.0)
No	39000	19.8 (18.0-21.8)
Risk for food insecurity^h^		
Yes	67000	35.4 (30.4–40.8)
No	123000	64.6 (59.2–69.6)

*CI* Confidence interval

Confidence intervals are a measure of estimate precision: the wider the CI, the more imprecise the estimate

Data are weighted to the NYC public high school student population and are not age-adjusted. Weighted population estimates are rounded to the nearest 1000

For the purpose of this publication, Latino/a includes persons of Hispanic, Latino, or Spanish origin, as identified by the survey question “Are you Hispanic or Latino?” and regardless of race. Black, White, Asian, and Another race categories excluded those who identified as Latino/a. Another race includes students who identified as American Indian, Native Hawaiian/Other Pacific Islander, or Multi-racial

^a^ Almost every day for > = 2 weeks in a row so that they stopped doing some usual activities, ever during the 12 months before the survey

^b^ This variable included the two questions: During the past 7 days, how many times did you eat fruit? (Do not count fruit juice.) and During the past 7 days, how many times did you eat vegetables such as green salad, carrots, green beans, or other vegetables? (Do not count potatoes.)

^c^ This variable included the two questions: During the past 7 days, how many times did you drink a can, bottle, or glass of soda or pop, such as Coke, Pepsi, or Sprite? (Do not count diet soda or diet pop.) and During the past 7 days, how many times did you drink other sugar-sweetened drinks such as sports drinks, energy drinks, fruit punch, fruit-flavored drinks, or sugar-sweetened teas? (Do not count sugar-free drinks.)

^d^ During the 7 days before the survey

^e^ Such as sausage, bacon, hot dogs, or cold cuts, during the 7 days before the survey

^f^ In any kind of physical activity that increased their heart rate and made them breathe hard some of the time any day during the 7 days before the survey

^g^ Did not get 8 or more hours of sleep (on an average school night)

^h^ This variable included two questions: During the past 12 months, how often did you worry that food at home would run out before your family got money to buy more? and During the past 12 months, how often did the food that your family bought run out and there was no money to buy more?

Regarding dietary behaviors, 9.5% of students did not eat vegetables or fruit, 30.6% drank a sugary drink one or more times per day, 20.4% did not eat breakfast, and 12.4% ate processed meat one or more times per day. Approximately one in five students (20.1%) did not participate in at least 60 min of physical activity and most students got insufficient sleep (80.2%). More than one third of students were at risk for food insecurity (35.4%).

Table 2 presents the prevalence estimates and bivariate differences in self-reported depressive symptoms, dietary behaviors, lifestyle habits, and food insecurity risk by race and ethnicity. Latino/a students (41.2%) had the highest prevalence of self-reported depressive symptoms followed by students of another race (38.6%), White students (33.0%), Asian students (31.4%), and Black students (30.7%). For dietary behaviors, there were significant associations between race and ethnicity with not eating vegetables or fruit, drinking a sugary drink one or more times a day, and not eating breakfast, with similar patterns seen across racial and ethnic groups. Black students and Latino/a students had the highest prevalences of not eating vegetables or fruit (16.4% and 9.8% respectively), drinking a sugary drink one or more times per day (32.4% and 33.7% respectively), and not eating breakfast (22.9% and 22.5% respectively). Whereas Asian students consistently had the lowest prevalence across these dietary behaviors (3.2%^1^, 23.4%, and 14.7% respectively). There was no association between race and ethnicity and eating processed meat one or more times per day (*p* = 0.774), indicating that prevalences were similar across racial and ethnic groups.

**Table 2:** Mental health outcome, dietary behaviors, lifestyle habits, and food insecurity risk by race and ethnicity among NYC public high school students

	White (*n* = 845)	Black (*n* = 1731)	Latino/a (*n* = 3531)	Asian (*n* = 1321)	Another race (*n* = 615)	*P*
	Weighted % (95% CI)	Weighted % (95% CI)	Weighted % (95% CI)	Weighted % (95% CI)	Weighted % (95% CI)	
Mental health outcome						
Self-reported depressive symptoms^a^	33.0 (28.0-38.3)	30.7 (23.6–38.7)	41.2 (38.8–43.7)	31.4 (28.6–34.3)	38.6 (34.2–43.2)	< 0.001
Dietary behaviors						
Did not eat fruit or vegetables^b^	5.5 (3.5–8.5)	16.4 (12.0-21.9)	9.8 (7.7–12.4)	3.2* (1.7–5.8)	8.7 (6.1–12.2)	< 0.001
Drank a sugary drink one or more times per day^c^	27.3 (23.6–31.2)	32.4 (25.4–40.2)	33.7 (31.2–36.3)	23.4 (20.2–27.0)	25.9 (21.4–30.9)	< 0.001
Did not eat breakfast^d^	18.6 (14.7–23.2)	22.9 (19.5–26.8)	22.5 (20.2–24.9)	14.7 (12.0-17.9)	20.3 (16.9–24.1)	0.007
Ate processed meat one or more times per day^e^	11.6 (8.5–15.5)	12.7 (9.3–17.1)	12.9 (11.1–15.0)	10.9 (8.6–13.7)	11.5 (8.8–15.0)	0.774
Lifestyle habits						
Did not participate in at least 60 min of physical activity^f^	11.7 (8.8–15.4)	28.1 (19.6–38.5)	21.0 (18.1–24.3)	15.3 (13.0–18.0)	19.7 (15.4–24.8)	0.001
Insufficient sleep^g^	78.4 (73.4–82.6)	79.8 (76.1–83.1)	80.1 (77.0-82.9)	81.3 (77.2–84.8)	78.7 (74.5–82.3)	0.756
Risk for food insecurity^h^	18.8 (13.9–24.9)	53.8* (41.2–66.0)	39.3 (35.1–43.7)	17.9 (13.5–23.4)	34.6 (29.0-40.7)	< 0.001

*CI* Confidence interval

Confidence intervals are a measure of estimate precision: the wider the CI, the more imprecise the estimate

Data are weighted to the NYC public high school population and not age-adjusted

For the purpose of this publication, Latino/a includes persons of Hispanic, Latino, or Spanish origin, as identified by the survey question “Are you Hispanic or Latino?” and regardless of race. Black, White, Asian, and Another race categories excluded those who identified as Latino/a. Another race includes students who identified as American Indian, Native Hawaiian/Other Pacific Islander, or Multi-racial

* Estimates should be interpreted with caution. Estimate's Relative Standard Error (a measure of estimate precision) is greater than 30%, or the 95% Confidence Interval half-width is greater than 10 or the sample size is too small, making the estimate potentially unreliable

^a^ Almost every day for > = 2 weeks in a row so that they stopped doing some usual activities, ever during the 12 months before the survey

^b^ This variable included the two questions: During the past 7 days, how many times did you eat fruit? (Do not count fruit juice.) and During the past 7 days, how many times did you eat vegetables such as green salad, carrots, green beans, or other vegetables? (Do not count potatoes.)

^c^ This variable included the two questions: During the past 7 days, how many times did you drink a can, bottle, or glass of soda or pop, such as Coke, Pepsi, or Sprite? (Do not count diet soda or diet pop.) and During the past 7 days, how many times did you drink other sugar-sweetened drinks such as sports drinks, energy drinks, fruit punch, fruit-flavored drinks, or sugar-sweetened teas? (Do not count sugar-free drinks.)

^d^ During the 7 days before the survey

^e^ Such as sausage, bacon, hot dogs, or cold cuts, during the 7 days before the survey

^f^ In any kind of physical activity that increased their heart rate and made them breathe hard some of the time any day during the 7 days before the survey

^g^ Did not get 8 or more hours of sleep (on an average school night)

^h^ This variable included two questions: During the past 12 months, how often did you worry that food at home would run out before your family got money to buy more? and During the past 12 months, how often did the food that your family bought run out and there was no money to buy more?

For lifestyle habits, there was a significant association between race and ethnicity and not participating in at least 60 min of physical activity. The highest prevalences were also observed among Black students (28.1%) and Latino/a students (21.0%); however, the lowest prevalence was seen among White students (11.7%). There was no association between race and ethnicity and insufficient sleep (*p* = 0.756), indicating that prevalences were similar across racial and ethnic groups. Lastly, there was an association between race and ethnicity and food insecurity risk. Consistent with patterns observed for dietary behaviors, Black students (53.8%^1^ ) and Latino/a students (39.3%) had the highest prevalence of being at risk for food insecurity, whereas Asian students (17.9%) had the lowest.

**Fig. 1:**
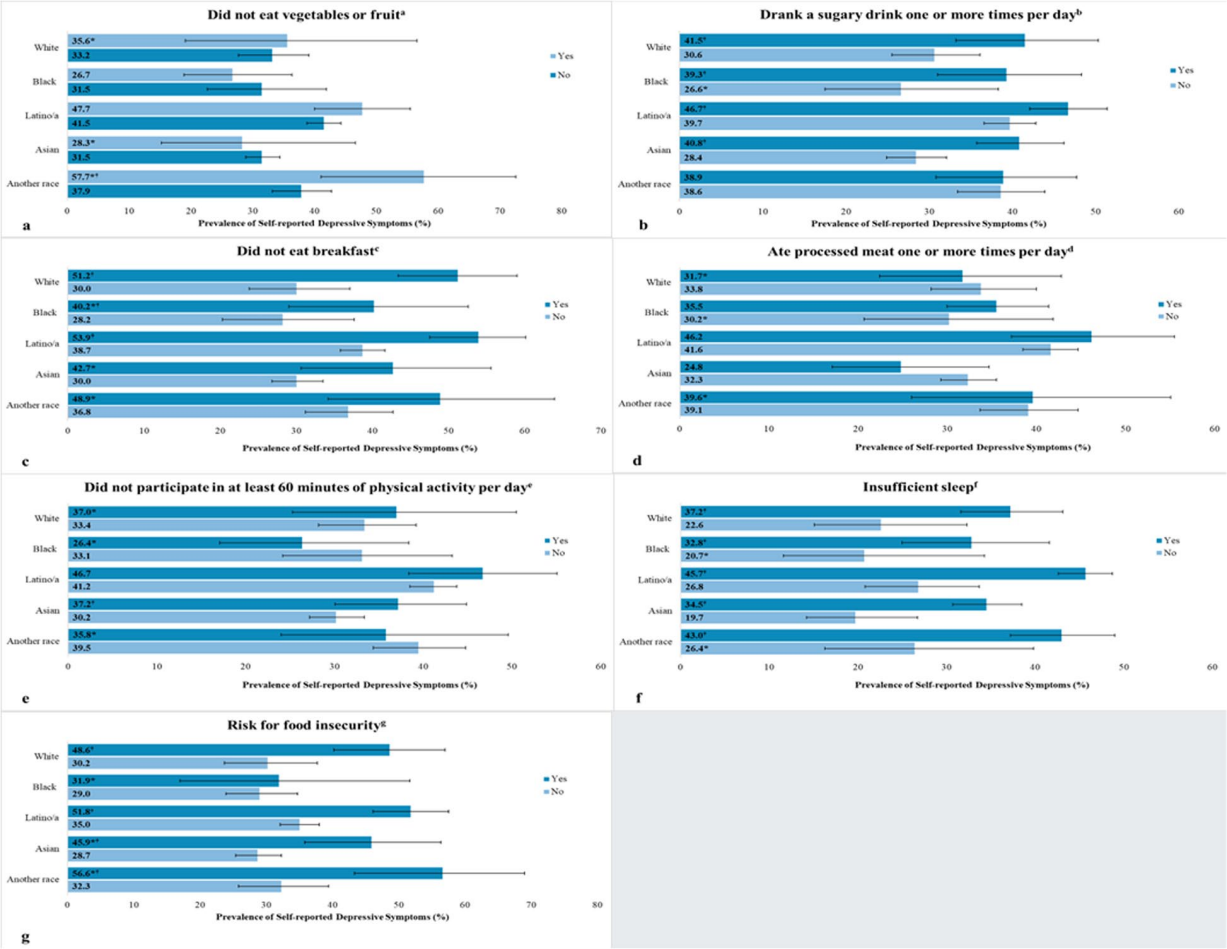
Bivariate associations between dietary behaviors, lifestyle habits, and food insecurity risk with self-reported depressive symptoms within racial and ethnic groups. CI= Confidence interval. Confidence intervals are a measure of estimate precision: the wider the CI, the more imprecise the estimate. Data are weighted to the NYC public high school population and are not age-adjusted. Clustered bar graphs show the prevalence of self-reported depressive symptoms within each racial and ethnic group for each exposure: (**a**) did not eat vegetables or fruit, (**b**) drank a sugary drink one or more times per day, (**c**) did not eat breakfast, (**d**) ate processed meat one or more times per day, (**e**) did not participate in at least 60 min of physical activity per day, (**f**) insufficient sleep, and (**g**) risk for food insecurity. Error bars indicate 95% confidence intervals. For the purpose of this publication, Latino/a includes persons of Hispanic, Latino, or Spanish origin, as identified by the survey question “Are you Hispanic or Latino?” and regardless of race. Black, White, Asian, and Another race categories excluded those who identified as Latino/a. Another race includes those who identified as American Indian, Native Hawaiian/Other Pacific Islander, or Multi-racial. * Estimates should be interpreted with caution. Estimate’s Relative Standard Error (a measure of estimate precision) is greater than 30%, or the 95% Confidence Interval half-width is greater than 10 or the sample size is too small, making the estimate potentially unreliable. ^a^ This variable included the two questions: During the past 7 days, how many times did you eat fruit? (Do not count fruit juice.) and During the past 7 days, how many times did you eat vegetables such as green salad, carrots, green beans, or other vegetables? (Do not count potatoes.) ^b^ This variable included the two questions: During the past 7 days, how many times did you drink a can, bottle, or glass of soda or pop, such as Coke, Pepsi, or Sprite? (Do not count diet soda or diet pop.) and During the past 7 days, how many times did you drink other sugar-sweetened drinks such as sports drinks, energy drinks, fruit punch, fruit-flavored drinks, or sugar-sweetened teas? (Do not count sugar-free drinks.) ^c^ During the 7 days before the survey ^d^ Such as sausage, bacon, hot dogs, or cold cuts, during the 7 days before the survey ^e^ In any kind of physical activity that increased their heart rate and made them breathe hard some of the time any day during the 7 days before the survey ^f^ Did not get 8 or more hours of sleep (on an average school night) ^g^ This variable included two questions: During the past 12 months, how often did you worry that food at home would run out before your family got money to buy more? and During the past 12 months, how often did the food that your family bought run out and there was no money to buy more? ^†^ Denotes a statistically difference between students who responded yes vs. no within the same racial and ethnic group (*p* < 0.05)

Figure 1a-g displays prevalence estimates and bivariate associations between dietary behaviors, lifestyle habits, and food insecurity risk with self-reported depressive symptoms within racial and ethnic groups. Within racial and ethnic groups, associations between self-reported depressive symptoms with dietary behaviors varied by behavior. Not eating vegetables or fruit was associated with self-reported depressive symptoms only among students of another race, with a higher prevalence among those who did not eat them (57.7%^1^ vs. 37.9%). In contrast, among White, Black, Latino/a and Asian students, those who drank a sugary drink one or more times per day were more likely to report self-reported depressive symptoms compared to those who did not. A similar pattern was observed for not eating breakfast, with students who did not eat breakfast more likely to report self-reported depressive symptoms; however, this association was seen only among White, Black, and Latino/a students. There was no association between eating processed meat one or more times per day and self-reported depressive symptoms by race and ethnicity.

Regarding lifestyle habits, the association between not participating in at least 60 min of physical activity and self-reported depressive symptoms was observed only among Asian students, with non-participants more likely to report self-reported depressive symptoms (37.2% vs. 30.2%). Sleep duration was the only measure consistently associated with self-reported depressive symptoms across all racial and ethnic groups, with higher prevalence seen among students who got insufficient sleep compared with their peers in the same racial and ethnic group who got sufficient sleep. Lastly, being at risk for food insecurity demonstrated a pattern similar to sleep duration; however, the association with self-reported depressive symptoms was not observed among Black students.

**Fig. 2:**
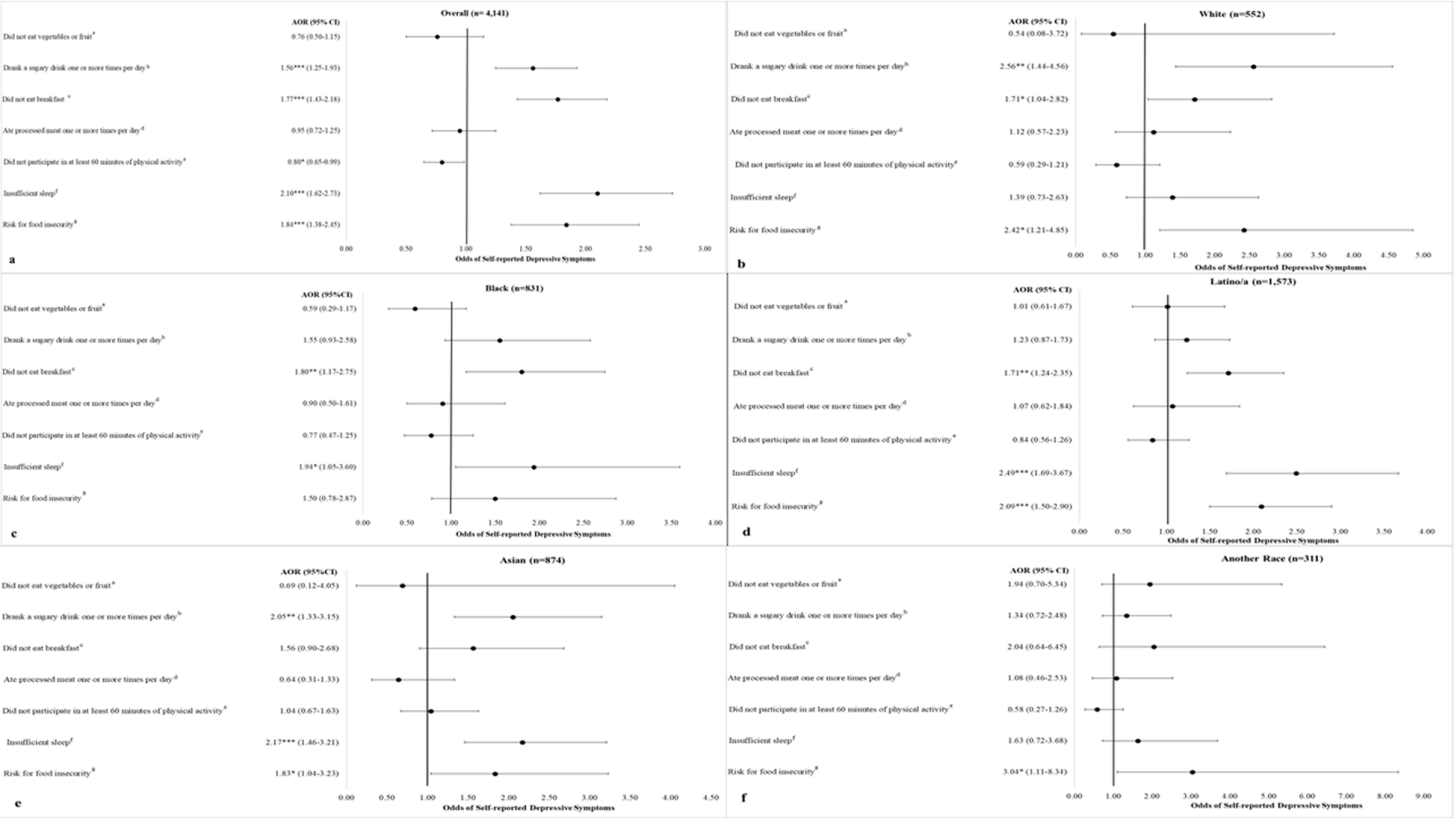
Associations between dietary behaviors, lifestyle habits, and food insecurity risk with self-reported depressive symptoms within racial and ethnic groups. CI= Confidence interval; AOR= Adjusted odds ratios. Data is not age-adjusted. Forest plots present adjusted odds ratios from models adjusted for age group, sex, borough of residence, sexual identity, and gender identity The overall model is additionally adjusted for race and ethnicity. Results are shown for overall population (Fig. 2**a**) and stratified by race and ethnicity (Fig. 2**b**-**f**), with each panel representing a distinct racial/ethnic group. For the purpose of this publication, Latino/a includes persons of Hispanic, Latino, or Spanish origin, as identified by the survey question “Are you Hispanic or Latino?” and regardless of race. Black, White, Asian, and Another race categories excluded those who identified as Latino/a. Another race includes those who identified as American Indian, Native Hawaiian/Other Pacific Islander, or Multi-racial. ^a^ This variable included the two questions: During the past 7 days, how many times did you eat fruit? (Do not count fruit juice.) and During the past 7 days, how many times did you eat vegetables such as green salad, carrots, green beans, or other vegetables? (Do not count potatoes.) ^b^ This variable included the two questions: During the past 7 days, how many times did you drink a can, bottle, or glass of soda or pop, such as Coke, Pepsi, or Sprite? (Do not count diet soda or diet pop.) and During the past 7 days, how many times did you drink other sugar-sweetened drinks such as sports drinks, energy drinks, fruit punch, fruit-flavored drinks, or sugar-sweetened teas? (Do not count sugar-free drinks.) ^c^ During the 7 days before the survey ^d^ Such as sausage, bacon, hot dogs, or cold cuts, during the 7 days before the survey ^e^ In any kind of physical activity that increased their heart rate and made them breathe hard some of the time any day during the 7 days before the survey ^f^ Did not get 8 or more hours of sleep (on an average school night) ^g^ This variable included two questions: During the past 12 months, how often did you worry that food at home would run out before your family got money to buy more? and During the past 12 months, how often did the food that your family bought run out and there was no money to buy more? *p < 0.05, ** *p* < 0.010, ***p < 0.001

Figure 2a-f focused on the independent effects of dietary behaviors, lifestyle habits, and food insecurity risk on self-reported depressive symptoms after adjusting for all demographics. For the overall population, students who drank a sugary drink one or more times per day, did not eat breakfast, got insufficient sleep, or were at risk for food insecurity had greater odds of self-reported depressive symptoms compared with students who did not drink a sugary drink one or more times per day, ate breakfast, got sufficient sleep, or were not at risk for food insecurity, respectively. However, students who did not participate in physical activity had 20% lower odds of self-reported depressive symptoms compared to those that did participate in physical activity. There was no association between not eating vegetables or fruit and eating processed meat with self-reported depressive symptoms.

Stratified models showed varying results, with no consistent associations observed habits, or risk for food insecurity across all racial and ethnic groups. Compared to students who did not drink a sugary drink one or more times per day, among White students who did had 2.56 higher odds of self-reported depressive symptoms (95% CI 1.44–4.56), while among Asian students who drank a sugary drink one or more times per day had twice the odds of self-reported depressive symptoms (AOR 2.05; 95% CI 1.33–3.15). No significant associations were observed among Black, Latino/a, and students of another race. There was a significant association between not eating breakfast and self-reported depressive symptoms. Compared with students who ate breakfast, nearly twice the odds of self-reported depressive symptoms were observed among White students (AOR:1.71; 95% CI 1.04–2.82), Black students (AOR: 1.80; 95% CI 1.17–2.75), and Latino/a students (AOR 1.71; 95% CI 1.24–2.35) who did not eat breakfast.

Sleep duration also exhibited a variable pattern of association with self-reported depressive symptoms, with nearly twice the odds observed among Black students (AOR 1.94; 95% CI 1.05–3.60), 2.49 times the odds among Latino/a students (95% CI 1.69–3.67), and 2.17 times the odds among Asian students (95% CI 1.46–3.21) who reported insufficient sleep compared with those who reported sufficient sleep. Compared with students who were not at risk for food insecurity, students who were at risk had higher odds of self-reported depressive symptoms across all racial and ethnic groups, expect Black students.

Notably, among students of another race, food insecurity risk was the only significant association with self-reported depressive symptoms, with those at risk of food insecurity having 3 times greater odds of self-reported depressive symptoms compared with their peers who were not at risk. This finding highlights a distinct pattern within this group. However, although these associations were statistically significant some estimates showed moderate imprecision, reflecting uncertainty in the exact magnitude of the effects (i.e., confidence intervals suggested that the true effect size ranged from medium to large). However, the direction of the associations remained consistent across comparisons, strengthening the overall conclusions.

## Discussion

Using NYC YRBS data, this cross-sectional analysis explored the association of dietary behaviors, lifestyle habits, and food insecurity risk with self-reported depressive symptoms among NYC public high school students by race and ethnicity. Our paper revealed some unexpected findings regarding the association between key lifestyle variables and self-reported depressive symptoms.

Our findings demonstrated that for the overall population, drinking sugary drinks, not eating breakfast, getting insufficient sleep, and being at risk for food insecurity were associated with greater odds of self-reported depressive symptoms. An unexpected finding was that not participating in physical activity was associated with lower odds of self-reported depressive symptoms for the overall population. Additionally, physical activity was not associated with self-reported depressive symptoms within the race-stratified models. Previous research on the association between physical activity and adolescent mental health have been mixed with studies suggesting either a positive association or no difference between physically active and non-physically active adolescents in mental health outcomes [76, 77]. However, there is no current evidence supporting our current findings of lower odds of self-reported depressive symptoms. Our unexpected findings may reflect the measurement limitations attributed to self-reported survey data such as recall bias, misclassification, and residual confounding. Therefore, this finding should be interpreted with caution and further research into the relationship between mental health and physical activity is warranted.

Findings also indicated that Black and Latino/a students consumed sugary drinks at higher rates compared to White students, Asian students, and students of another race, which aligns with existing literature that sugary drink consumption is higher among Black and Hispanic youth [78] and is reflective of a beverage industry that has disproportionately invested financial and social capital into communities of color [79]. However, despite lower reported rates of sugary drink consumption among White and Asian students, sugary drink consumption was significantly associated with greater odds of self-reported depressive symptoms among White and Asian students only. The absence of an association with self-reported depressive symptoms among Black students, Latino/a students, and students of another race is notable and warrants further research on other factors that may have a greater influence and/or impact on the association with sugary drink consumption. Furthermore, not eating breakfast was associated with greater odds of self-reported depressive symptoms for White, Black, and Latino/a students at similar effect sizes and was not significantly associated among Asian students or students of another race. Our findings are consistent with previous research using national YRBS data that reported associations between self-reported depressive symptoms with not eating breakfast [7].

Insufficient sleep was associated with greater odds of self-reported depressive symptoms for Black, Latino/a, and Asian students, but not White students or students of another race, with the greatest effect size seen for Latino/a students. Our results corroborate national research showing that students who got insufficient sleep were more likely to report worse mental health compared to students who got sufficient sleep [80, 81]. Racial and ethnic minorities are also at increased risk of insufficient sleep compared to their White peers [82]. This is particularly concerning because our analysis found that over three-quarters of NYC students in every race and ethnic demographic reported insufficient sleep.

Food insecurity risk was not associated with greater odds of self-reported depressive symptoms among Black students, despite Black students having the highest rates of risk of food insecurity (53.8%^1^) among all demographic groups, which is consistent with national data [83]. Risk of food insecurity was associated with greater odds of self-reported depressive symptoms among White, Latino/a, Asian students, and students of another race, with the greatest effect sizes seen among students of another race. Another race category is heterogenous and included racial and ethnic groups that have been found to experience higher levels of food insecurity, depression, and other mental health outcomes compared to other race categories [84–86] Research has found an association between food insecurity and mental health, finding that students experiencing food insecurity are more likely to report depressive symptoms, seriously consider suicide, and exhibit externalizing/internalizing behaviors (e.g., directing problematic energy outwards and towards oneself, respectively) [87, 88]. While racial and ethnic minorities disproportionately experience food insecurity at higher rates than their White peers [83], it is worth exploring both why the association between food insecurity risk and self-reported depressive symptoms was greater for White students than students of other race and ethnicities and why risk for food insecurity was not associated with increased odds of self-reported depressive symptoms among Black students .

It is important to acknowledge other mechanisms that may be impacting youth mental health that are not captured in our paper. The cross-sectional associations we found may be related to social determinants of mental health, including income, education, employment, safe and stable housing, access to health care, and discrimination and racism. Low-income levels can make it difficult to access mental health services and there are racial and ethnic inequities in access to mental health services among youth [89, 90]. Housing instability and homelessness can increase the risk of psychiatric conditions like depression, anxiety, and psychosis [91, 92]. Further, people who report experiencing discrimination in daily life are more likely to report adverse effects from worry or stress and feelings of loneliness, anxiety, and depression [93]. Repeated exposure to these social determinants of health can affect people’s opportunity to maintain good mental health. Therefore, these upstream factors may be much stronger predictors of self-reported depressive symptoms than lifestyle or dietary habits. This may explain the lack of association of food insecurity risk with self-reported depressive symptoms among Black students, but more research is needed. Given the missing data and cross-sectional design, these findings should be interpreted with caution, highlighting the need for further research to understand the complex interacting nature of sleep, diet, physical activity, and mental health.

Social determinants of health can be addressed through policies and programs that address basic needs such as universal school meals and breakfast-after-the-bell programs, universal healthcare, robust coverage for the Supplemental Nutrition Assistance Program and Medicaid, increased access to federal income support, and increasing affordable housing opportunities [94]. At the local level, NYC implements a wide range of initiatives that work to address upstream drivers of inequitable health outcomes, as well as school-based interventions that promote adolescent physical and mental well-being, some of which are described below.

### NYC citywide initiatives

In March 2023, NYC launched *Care, Community, Action: A Mental Health Plan for NYC*, a comprehensive plan focusing on communities with the highest mental health needs which included children, youth, and families [95]. The plan outlined goals and strategies including timely and accessible mental health care, improved access to prevention interventions, increased awareness and understanding of children’s mental health, and the creation of environments that equitably support good mental health [95]. Following in January 2025, the NYC Health Department released *Addressing Unacceptable Inequities: A Chronic Disease Strategy for New York City*, a multiagency strategy that addresses the root causes of chronic diseases such as heart disease, diabetes, and screenable cancers and outlines interventions that support longer, healthier lives for New Yorkers [44]. The NYC Health Department also prioritizes high-need areas by investing in Bureaus of Neighborhood Health in North and Central Brooklyn, East Harlem, and the South Bronx and in the neighborhoods across the city with a high percentage of health and socioeconomic disparities, to anchor our place-based strategies with neighborhood-specific services to reduce health inequities and improve health outcomes [96, 97] .

### NYC school-based interventions

NYC Public Schools has a written wellness policy in place, *Empowering Strong and Healthy Students: The New York City Public Schools (NYCPS) School Wellness Policy.* The policy ensures students have access to physical activity, free and healthy meals and snack choices, nutrition and health education and services, and mental health programs [98]. NYCPS and NYC Health Department have also established the joint initiative, School Mental Health (SMH) whose mission is to ensure that every child has high quality mental health resources [99]. SMH has partnered with community-based organizations that operate in schools to implement mental health initiatives [99]. Additionally in March 2025, NYC Health + Hospitals announced the opening of 16 mental health clinics in NYCPS. Clinics will provide individual, family, or group therapy for students, along with consultations, trainings, and workshops for teachers and school staff [100].

### Strengths and limitations

Our cross-sectional paper is strengthened by using NYC YRBS data, which is representative of NYC public high school students and offers details on this population not possible in national data sets. Data on the opinions and lived experiences of adolescent populations are not as common as those available for adults, so this paper offers an insight not often captured. Despite these strengths, this study is not without limitations.

First, logistic regression analyses were conducted using a complete case approach which resulted in a substantial reduction in sample size. This reduction in sample size may also decrease survey efficiency, precision, and statistical power [101, 102]. Missingness differed across demographics and exposure variables, with higher levels observed among marginalized groups including Latino/a, Black, and gender minority students and students at risk for food insecurity, suggesting that the data were not missing completely at random. As a result, the exclusion of these participants may have led to underrepresentation and could bias estimates [101]. Analyses also involved stratification and multiple comparisons which can increase the probability of false positives (Type 1 errors) [103]. Together, these factors may reduce the study’s power to detect disparities. Second, the YRBS does not measure economic status, hindering our ability to assess a critical socioeconomic characteristic often associated with both mental health and food security which may contribute to residual confounding in the observed associations. Additionally, the absence of controlling for other relevant measures, associated physical activity and mental health, may explain the unexpected finding observed in this study. Third, despite the YRBS being a representative sample, it is only representative of NYC youth who attend public, charter, and vocational high schools; thus, we cannot generalize to all youth in NYC.

Fourth, the YRBS is not a longitudinal study in that the same youth continue to participate over time. Exposures and outcomes were measured concurrently, and we were able to look at the data only from a cross-sectional perspective. Consequently, temporal relationships cannot be determined, causality cannot be inferred, and reverse causation remains a possibility. Thus, observed associations between sleep, dietary behaviors, food insecurity risk, physical activity, and mental health may reflect multidirectional relationships. Fifth, it is important to keep in mind that the YRBS uses single item self-reported data. Single item measures shorten survey length, minimize participant burden, and are less time-consuming to respondents; however they are not designed to capture the full range of constructs like multi-item measures [104]. Multi-item measures allow for a more in-depth analysis of underlying factors that contribute to the outcome investigated [104]. The use of self-reported data may introduce recall bias and social desirability bias. The YRBS questions used in this study had varying recall periods (e.g. in the last 30 days vs. in the last 12 months) which can impact accuracy of responses, as well as the ability for participants to recall information over wide timeframes. An inaccurate recall of events can potentially overestimate or underestimate the associations found in the study. Social desirability bias occurs when respondents describe themselves positively, rather than truthfully and accurately, potentially leading to overreporting of desirable characteristics and the underreporting of undesirable ones [105]. Prior research on social desirability bias and self-reported dietary behaviors and physical activity in adolescents demonstrated biases towards healthier behaviors [106, 107].

Sixth, our exposures were binary with two exposures being unvalidated composite measures. The use of the non-validated composite measures for sugary drink and vegetable and fruit consumption raises concerns of their validity and reliability. Composite variables may also change the strength of the relationship with the outcome variables, lose information, and reduce statistical power [108]. Similar disadvantages may be observed in our analytic approach, by using binary and dichotomized variables. While using dichotomized variables simplifies interpretation, valuable information can be lost when collapsing multiple categories which can obscure nuanced relationships [109]. Misclassification can also arise if participants have been incorrectly categorized and may be differential or non-differential depending on whether classification errors vary across participants. Both forms of misclassification can bias estimates, leading to the overestimation or underestimation of associations [110].

## Conclusion

Our paper found that dietary behaviors, lifestyle habits, and food insecurity risk are associated with self-reported depressive symptoms among NYC public high school students and that there are differences across race and ethnicity. The high prevalence of reported food insecurity risk and insufficient sleep indicates that these are key areas for interventions as both factors are linked to poorer mental health outcomes. However, due to the cross-sectional nature of this study, longitudinal research focused on these interventions is necessary to determine if addressing these factors will lead to improved mental health outcomes. Addressing these factors, alongside upstream social determinants is necessary for promoting mental health equity among NYC public high school students. As our paper was cross-sectional, more research is needed on how other social determinants of health may be influencing the interaction between these variables and self-reported depressive symptoms. Nevertheless, our findings support and underscore the importance of sustaining and enhancing existing NYC policies and initiatives that promote healthy diet and lifestyle behaviors, food access, and mental health.

## Supplementary Information

Supplementary Material 1.

## Data Availability

The dataset analyzed during this current study is not publicly available due to the internal approval process. If possible, study analysis code can be provided upon reasonable request to the corresponding author.

## Footnotes

1 Estimates with a relative standard error greater than 30%, the 95% confidence interval half width was greater than 10, or the sample size was too small were considered unreliable and were flagged to be interpreted with caution.

## Abbreviations

US: United States
NYC: New York City
SPD: Serious psychological distress
YRBS: Youth Risk Behavior Survey
CDC: Center for Disease Control and Prevention
NYCPS: New York City Public Schools
VIF: Variance inflation factor
AOR: Adjusted odds ratio
CI: Confidence interval
SMH: School Mental Health
