# Supplementary material for: Dietary behaviors, lifestyle habits, food insecurity risk and depressive symptoms among New York City adolescents: a cross-sectional study
Source: BMC Pediatr. 2026 Apr 23;26:535. doi: 10.1186/s12887-026-06819-1 (PMC13244642; doi:10.1186/s12887-026-06819-1)
Supplement: Supplementary file 1 — Supplementary Material 1. [file 12887_2026_6819_MOESM1_ESM.docx]

| **Variable** | **Question** | **Response Options** | **Analytic Coding** |
| --- | --- | --- | --- |
| **Mental Health** | | | |
| Self-reported depressive symptoms | During the past 12 months, did you ever feel so sad or hopeless almost every day for two weeks or more in a row that you stopped doing some usual activities? | Yes, No | Yes vs. No |
| **Dietary Behaviors** | | | |
| Did not eat vegetables or fruit | Based on the two questions: (1) During the past 7 days, how many times did you eat fruit? (Do not count fruit juice) and (2) During the past 7 days, how many times did you eat vegetables such as green salad, carrots, green beans, or other vegetables? (Do not count potatoes) | I did not eat fruits/vegetables during the past 7 days, 1 to 3 times during the past 7 days, 4 to 6 times during the past 7 days, 1 time per day, 2 times per day, 3 times per day, 4 or more times per day | Summed both items to determine vegetable and fruit consumption and responses were dichotomized as did not eat vegetables or fruit vs. ate vegetables or fruit |
| Drank a sugary drink one or more times a day | Based on the two questions: (1) During the past 7 days, how many times did you drink a can, bottle, or glass of soda or pop, such as Coke, Pepsi, or Sprite? (Do not count diet soda or diet pop.) and (2) During the past 7 days, how many times did you drink other sugar-sweetened drinks such as sports drinks, energy drinks, fruit punch, fruit-flavored drinks, or sugar-sweetened teas? (Do not count sugar-free drinks). | I did not drink soda or pop/other sugar-sweetened drinks, 1 to 3 times during the past 7 days, 4 to 6 times during the past 7 days, 1 time per day, 2 times per day, 3 times per day, 4 or more times per day | Summed both items to determine sugary drink consumption and responses were dichotomized one or more times a day vs. less than one time per day |
| Did not eat breakfast | During the past 7 days, how many days did you eat breakfast? | 0 days, 1 day, 2 days, 3 days, 4 days, 5 days, 6 days, 7 days | 0 days vs. 1-7 days |
| Ate processed meat one or more times per day | During the past 7 days, how many times did you eat processed meat, such as sausage, bacon, hot dogs, or cold cuts? | I did not eat processed meats during the past 7 days, 1 to 3 times during the past 7 days, 4 to 6 times during the past 7 days, 1 time per day, 2 times per day, 3 times per day, 4 or more times per day | One or more times per day vs. less than one time per day |
|  |  |  |  |
| Risk for food insecurity | Based on the two questions: (1) During the past 12 months, how often did you worry that food at home would run out before your family got money to buy more? and (2) During the past 12 months, how often did the food that your family bought run out and there was no money to buy more? | Often, Sometimes, Never | Yes (Respondents had to respond Often or Sometimes to either questions) vs. No (Never) |
| **Lifestyle Habits** | | | |
| Did not participate in at least 60 minutes of physical activity | During the past 7 days, on how many days were you physically active for a total of at least 60 minutes per day (Add up all the time you spent in any kind of physical activity that increased your heart rate and made you breathe hard some of the time) | 0 days, 1 day, 2 days, 3 days, 4 days, 5 days, 6 days, 7 days | 0 days vs. 1-7 day |
| Insufficient sleep | On an average school night, how many hours of sleep do you get? | 4 hours or less, 5 hours, 6 hours, 7 hours, 8 hours, 9 hours, 10 or  more hours | Yes (7 hours or less) vs. No (8 hours or more) |
